# In Vitro Evaluation of Cocoa Pod Husk Pectin as a Carrier for Chronodelivery of Hydrocortisone Intended for Adrenal Insufficiency

**DOI:** 10.1155/2017/8284025

**Published:** 2017-12-24

**Authors:** Ofosua Adi-Dako, Kwabena Ofori-Kwakye, Mariam El Boakye-Gyasi, Samuel Oppong Bekoe, Samuel Okyem

**Affiliations:** ^1^Department of Pharmaceutics, Faculty of Pharmacy and Pharmaceutical Sciences, College of Health Sciences, Kwame Nkrumah University of Science and Technology (KNUST), Kumasi, Ghana; ^2^School of Pharmacy, University of Ghana, Legon, Ghana; ^3^Department of Pharmaceutical Chemistry, Faculty of Pharmacy and Pharmaceutical Sciences, College of Health Sciences, Kwame Nkrumah University of Science and Technology (KNUST), Kumasi, Ghana; ^4^Central Laboratory, Kwame Nkrumah University of Science and Technology (KNUST), Kumasi, Ghana

## Abstract

This study evaluated the* in vitro* potential of cocoa pod husk (CPH) pectin as a carrier for chronodelivery of hydrocortisone intended for adrenal insufficiency. FTIR studies found no drug-CPH pectin interactions, and chemometric analysis showed that pure hydrocortisone bears closer similarity to hydrocortisone in hot water soluble pectin (HWSP) than hydrocortisone in citric acid soluble pectin (CASP). CPH pectin-based hydrocortisone matrix tablets (~300 mg) were prepared by direct compression and wet granulation techniques, and the tablet cores were film-coated with a 15% HPMC formulation for timed release, followed by a 12.5% Eudragit® S100 formulation for acid resistance.* In vitro* drug release studies of the uncoated and coated matrix tablets in simulated gastrointestinal conditions showed that wet granulation tablets exhibit greater retardation of drug release in aqueous medium than directly compressed tablets. CASP showed greater suppression of drug release in aqueous medium than HWSP. Wet granulation HWSP-based matrix tablets coated to a final coat weight gain of ~25% w/w were optimized for chronodelivery of hydrocortisone in the colon. The optimized tablets exhibited a lag phase of ~6 h followed by accelerated drug release in the colonic region and have potential to control night time cortisol levels in patients with adrenal insufficiency.

## 1. Introduction

Chronodelivery is the drug delivery approach whereby drugs and other bioactive substances are released in the body based on the clinical needs of the patient. Drug release in chronodelivery varies with time and synchronizes with the circadian rhythm of diseases [1, 2]. Chronotherapeutics denotes a treatment approach where drug made available to the body is timed to match the rhythms of disease to optimize treatment outcomes and minimize side effects [1, 3]. The therapeutic outcomes of diseases such as bipolar disorder, rheumatoid arthritis, hypertension, and cancer which follow the circadian rhythm have been optimized through chronotherapy [4, 5].

Adrenal insufficiency is characterized by a disruption of the body's cortisol rhythm through inadequate cortisol production. The condition occurs in all age groups and is potentially life threatening [6]. Most people with cortisol deficiency requires life-long hydrocortisone replacement to enhance their quality of life [7]. Cortisol (hydrocortisone), which is a hormone produced by the adrenal cortex, plays a pivotal role in the functionality of the circulatory system; immune system; stress responses; fat, protein, and carbohydrate metabolism; and bone formation. In the body, cortisol is released in a diurnal rhythm synchronized to the natural cycles of day and night [8]. The circadian profile of cortisol peaks in the morning, dips at midnight, and rises again around 3 am [9, 10].

The management of adrenal insufficiency involves the replenishment of cortisol in the body to the normal physiological levels [6]. The use of oral immediate release hydrocortisone preparations is unable to replicate the cortisol rhythm leading to increased morbidity and mortality. Hydrocortisone has a short elimination half-life of about 1.5 h, and patients on such hydrocortisone products are unable to attain peak cortisol levels after an hour and wake up with very low undetectable levels of the drug [11, 12]. There is, therefore, a clinical need for more appropriate hydrocortisone dosage forms to provide adequate blood levels of the drug, ensure patient compliance, and improve treatment outcomes [9, 10].

To address the deficiencies of immediate release hydrocortisone preparations in the treatment of adrenal insufficiency, several oral modified release preparations which are intended to mimic the physiological cortisol rhythm have been developed. A once daily hydrocortisone tablet with biphasic release properties produced a circadian-based serum cortisol profile relative to the conventional hydrocortisone dosage form [13, 14]. Plenadren®, another once daily dual-release hydrocortisone formulation, produced cortisol concentrations in the afternoon but not in the evening and showed no overnight rise in cortisol blood levels. The result was that patients experienced long periods of low cortisol concentrations from late afternoon until the morning when they took the next dose [10, 15]. Chronocort® was also investigated as a delayed release hydrocortisone product to replicate the overnight rise in cortisol. A 30 mg dose of the product administered at 22:00 hours gave an overnight rise 4 h later, and pharmacokinetic modeling suggested the administration of a 20 mg dose at 23:00 hours and a 10 mg dose at 7:00 hours would replicate the normal cortisol rhythm [10, 16]. The latter strategies were however deemed impractical as their sleep would have to be interrupted in order for them to benefit. In addition, daytime fatigue and disorientation could result from the sleep fragmentation [9]. The use of controlled release preparations which provide constant drug release are generally inappropriate for the management of adrenal insufficiency as they could enhance the adverse effects of the drug and promote drug resistance.

A more practical solution to the challenge of managing adrenal insufficiency is the formulation of an oral delayed, timed-dependent release hydrocortisone preparation that would release the drug 6 h after administration when there is a reduction in cortisol levels and mimic the physiological cortisol rhythm. This preparation would ensure compliance of patients to therapy. The chronodelivery formulation approach adopted would involve the preparation of an oral delayed release, colon targeted hydrocortisone product. This product would comprise a drug matrix containing CPH pectin coated with pH-dependent and time-dependent polymers to achieve time control, site specificity acid resistance [17]. Pectins as naturally occurring polysaccharides, hydrate, gel, and swell in aqueous media and have been employed in numerous delayed, modified release, colon-specific, and chronotherapeutic drug formulations [18–22].

CPHs are the empty, environmental waste, pod shells which remain after the recovery of cocoa* (Theobroma cacao)* beans from cocoa fruit. CPHs constitute about 52–76% of the cocoa fruit and are a rich source of pectin [23–25]. Although considerable research has been devoted to the applications of pectins from commercial sources in modified and colonic drug delivery, little attention has been paid to pectins from* Theobroma cacao *pod husk. In the current study, we investigate the* in vitro* potential of CPH pectin as a carrier for the chronodelivery of hydrocortisone intended for the management of adrenal insufficiency.

## 2. Materials and Methods

### 2.1. Materials

Micronised hydrocortisone ~50 um, dibutyl phthalate, and acetone were obtained from Sigma Aldrich, USA. Eudragit S 100, sodium hydroxide and 0.1 N HCl were obtained from Fischer Scientific, USA. Polyethylene glycol 400 (PEG 400), talc, and magnesium stearate were obtained from Fischer Scientific, UK. Microcrystalline cellulose (Avicel® PH 101), sodium starch glycolate (Explotab®), dibasic calcium phosphate (Emcompress®), and hydroxypropylmethyl cellulose (Methocel® E50) were kind gifts from Colorcon, USA. Isopropyl alcohol (Contec® sterile 70% isopropanol) was purchased from Contec Inc., USA. All other chemicals used were of analytical grade. Fresh CPHs were obtained from ripe* Theobroma cacao *L. (family: Sterculiaceae) fruits harvested from an experimental plantation of the Cocoa Research Institute of Ghana (CRIG), Tafo. Pectin was extracted from the CPHs at ~50°C with hot water (HWSP) and 4% w/v hot aqueous citric acid (CASP) as reported elsewhere [23, 25]. The CPH pectins with a degree of esterification of 26.8% were freeze-dried (120 mbar at −41°C) and stored in aluminium foils in a desiccant at −4°C until used.

### 2.2. FTIR Drug-Excipient Compatibility Studies

Drug-excipient compatibility was evaluated with a Bruker FTIR spectrophotometer (Jos Hansen & Soehne GmbH, Hamburg, Germany) operating on Platinum ATR to obtain FTIR spectra in a wavelength range of 4000–400 cm^−1^ at 4 cm^−1^ resolution. The spectra for pure hydrocortisone, HWSP, CASP, and physical mixtures of hydrocortisone in HWSP (0.5 : 1) and hydrocortisone in CASP (0.5 : 1) were evaluated. The spectra for hydrocortisone, HWSP, or CASP and the physical mixtures of hydrocortisone and the CPH pectins were superimposed and the similarities and differences in the spectra were determined.

### 2.3. Chemometric Analysis of FTIR Spectra

Chemometric analysis of the FTIR spectra was conducted with the use of Unscrambler® X software (CAMO Scientific, Norway). The FTIR spectra were subjected to data pretreatment such as multiplicative scatter correction and baseline correction to remove much of the variation in the spectra due to noise from the instrument. Savitzky-Golay second derivative with 45 smoothing points of the spectra revealed most of the variation in the spectra. Principal component analysis (PCA) was performed on pretreated second derivative spectra of selected wavelength range by using three PCs and cross-validation, with Hotellin T2 outlier determination at 95% confidence interval. Hierarchical complete linkage cluster analysis with squared Euclidean distance was also conducted on pretreated second derivative spectra.

### 2.4. Preparation of Pectin-Based Hydrocortisone Tablet Formulations

Five different tablet formulations each containing ~10 mg hydrocortisone were prepared by either direct compression or wet granulation technique.  Table 1 shows the composition of the tablet formulations. Formulations F1–F3 were prepared by direct compression while formulations F4 and F5 were prepared by wet granulation. Formulation 1 was designed to achieve immediate drug release while formulations F2–F5 were intended for modified drug release and contained CPH pectin as the release modifier. In the preparation of formulations F1–F3, the various powders were individually screened through a number 18 mesh sieve (1000 *μ*m), weighed, and dry mixed in a V-blender (Cadmach Machinery, India) for 15 min. In the preparation of formulation F4, the amount of HWSP was weighed and dispersed in sufficient amount of hot water to form viscous dispersion. The rest of the powders were added to the dispersion by geometric dilution and the powder mass granulated into a wet mass to form compacts. The wet powder mass was passed through a number 18 mesh sieve (1000 *μ*m) and the granules dried at 40°C for 90 min. The procedure was repeated for formulation F5 which contains CASP. Dispersion of the pectin was prepared in sufficient hot water containing 4% w/v citric acid and used as granulating fluid. Magnesium stearate was added to formulations F4 and F5 and mixed in the V-blender for a further 5 min. The dry granules of the five powder formulations were stored in airtight containers for further analysis.

### 2.5. Compression of Pectin-Based Matrix Tablets

The five lubricated CPH pectin-based powder formulations in the size range 595–1000 *μ*m containing ~10 mg hydrocortisone were compressed into tablets with a nominal tablet weight of 300 mg using a Single Punch Carver Tablet Press (Carver Inc., Wabash IN, USA) fitted with a concave punch and die set. The tablets were stored for 24 h after compression for elastic recovery before analysis.

### 2.6. Postcompression Evaluation of Tablets

The mean tablet weight (±S.D, *n* = 3) was determined by weighing ten randomly sampled tablets from each batch individually on a precision balance (Mettler Toledo, USA). The mean diameter and thickness (±S.D, *n* = 3) of ten randomly sampled tablets were determined with a vernier caliper (Mitutoyo, Japan). The friability of tablets from each batch was determined with a friabilator (USP) (SOTAX AG, Switzerland). Tablets equivalent to ~6 g from each formulation were randomly selected and weighed on a precision balance. The tablets were placed in a friabilator and rotated at 100 revolutions. The tablets were removed, dedusted, and reweighed, and the difference in weight was expressed as a percentage of the initial weight. Tablet hardness (mean ± S.D, *n* = 3) was determined using a Monsanto hardness tester (Mumbai, India) and the force required to break up the tablets was noted. Disintegration test was conducted on the immediate release hydrocortisone tablet formulation F1 which contains a superdisintegrant. The other formulations being modified release were not designed to disintegrate in aqueous media. Seven hundred and fifty milliliters of distilled water was measured into each of the 1L beakers of the QC-21 Disintegration Test System (Hanson Research, California, USA), after which the equipment was equilibrated to 37 ± 0.5°C. Six tablets of F1 were placed in each of the transparent tubes of the first basket rack. The procedure was repeated for the second basket rack. Both racks were lowered and immersed in the disintegration fluid and the test was run. The time taken for the six tablets in a rack to disintegrate was noted as the disintegration time.

### 2.7. HPLC Assay of the Hydrocortisone Matrix Tablets

An isocratic, validated reversed-phase high performance liquid chromatography (RP-HPLC) method recently described by Adi-Dako et al. [26] was employed to assay the hydrocortisone tablets. The HPLC instrument was equipped with a model Spectra Series P100 isocratic pump, Rheodyne injector with a 20 *μ*l loop, ultraviolet-visible detector (PerkinElmer series 785A), and PowerChrom series 280 integrator. The RP-HPLC analysis was performed with a C18 column, 5 *μ*m, 4.6 × 150 mm JT Baker ODS with mobile phase of methanol/water/acetic acid (60 : 30 : 10, v/v), flow rate of 1 ml/min, injection volume of 20 *μ*l, methanol as diluent, and ultraviolet detection at 254 nm.

### 2.8. Tablet Film-Coating Process

Two coating formulations were prepared and used for film-coating the hydrocortisone matrix tablets. Coating formulation 1 (CF1) comprising dispersion of 15% w/v HPMC (Methocel E50) and 0.5% w/v polyethylene glycol (PEG 400) was prepared by dispersing 7.5 g of HPMC (Methocel E50) and 0.25 g of PEG 400 in 20 ml distilled water and stirred with a glass rod. The viscous dispersion formed was made up to 50 ml with distilled water and stored. Coating formulation 2 (CF2) comprising 12.5% w/v dispersion of Eudragit S100 in isopropyl alcohol and acetone (1 : 1), 3% w/v of talc, and 1% w/v dibutyl phthalate was prepared by dispersing 12.5 g of Eudragit S100 in 30 ml of isopropyl alcohol and acetone (1 : 1) and stirred with a glass rod. Three grams (3 g) of talc and 1 g of dibutyl phthalate were added and made up to 100 ml with isopropyl alcohol and acetone (1 : 1). The uncoated hydrocortisone matrix tablet cores F4 and F5 were coated with the HPMC-based coating formulation (CF1) to a tablet weight gain of ~5% w/w, followed by the Eudragit-based coating formulation (CF2) to a final tablet weight gain of ~25% w/w, using the dip coating technique in a fume hood [17, 27, 28]. The film-coated tablets containing HWSP and CASP were designated as formulations F6 and F7, respectively.

### 2.9. *In Vitro* Dissolution of Hydrocortisone Tablet Formulations

The release of hydrocortisone from the uncoated and film-coated hydrocortisone matrix tablets was monitored using a USP Dissolution Tester Apparatus II (Hansen Research Corp., USA) with the paddle speed set at 50 rpm and temperature of 37 ± 0.5°C. One liter portions of simulated gastrointestinal fluids, without enzymes, were used as the dissolution media. Dissolution of the uncoated tablet formulations was undertaken separately in simulated gastric fluid (pH 1.2) and simulated small intestinal fluid (pH 6.8) for 60 min while the dissolution of the two film-coated tablet formulations was carried out in simulated gastric fluid (pH 1.2) for 2 h, replaced with simulated intestinal fluid (pH 6.8) for 3 h, and replaced again with simulated colonic fluid (pH 7.4, without enzymes) for 6 h. All the simulated gastrointestinal fluids were prepared according to USP (2013) [29] protocols. At specified time intervals, 5 ml aliquots were drawn for analysis and replaced with the same volume of dissolution medium. The drawn aliquots were centrifuged (Eppendorf centrifuge, model 5810R, Eppendorf AG, Hamburg, Germany) for 6 min at 4000 rpm and hydrocortisone levels were detected with a BioTek Microplate Reader using UV absorbance and analysed with Neosynergy software. Three replicate determinations were made for each tablet formulation.

### 2.10. Kinetics of Drug Release

The* in vitro* hydrocortisone release data was fitted into zero order, first order, Higuchi, Hixson-Crowell, and Korsmeyer-Peppas kinetic models. The kinetic model which produced the highest correlation coefficient (*R*^2^) for a drug release profile was considered to be the best fit for that profile.

### 2.11. Statistical Analysis

The drug release data of the five uncoated hydrocortisone matrix tablet formulations were subjected to one-way analysis of variance (ANOVA) followed by Newman-Keuls multiple comparison test while the release data of the two film-coated tablet formulations were compared using Student's *t*-test, both with GraphPad Prism version 5.00 for Windows (GraphPad Software, San Diego California, USA, https://www.graphpad.com/). Differences between tablet formulations were considered significant when *P* < 0.05.

## 3. Results and Discussion

### 3.1. Drug-Excipient Compatibility Studies and Chemometric Analysis of FTIR Spectra

**Figure 1:**
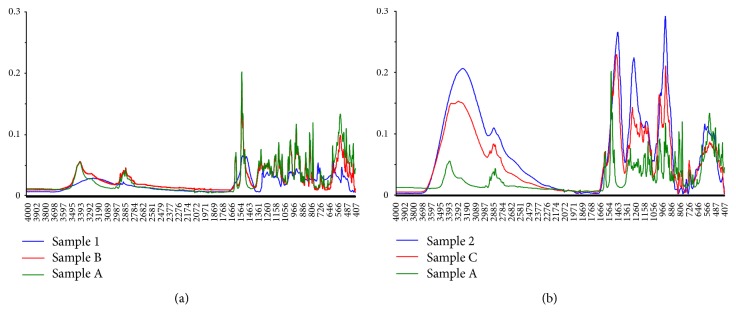
Superimposed FTIR spectra of (a) pure hydrocortisone (Sample A), HWSP (Sample 1), and physical mixture (0.5 : 1) of hydrocortisone in HWSP (Sample B); (b) pure hydrocortisone (Sample A), CASP (Sample 2), and physical mixture (0.5 : 1) of hydrocortisone in CASP (Sample C).

Drug-excipient compatibility studies are used to identify potential interactions between a drug and its excipients. Incompatibility between a drug and its excipient(s) can have adverse effects on the stability and bioavailability of the dosage form [30].  Figure 1(a) presents the superimposed FTIR spectra of pure hydrocortisone (Sample A), HWSP (Sample 1), and a (0.5 : 1) mixture of hydrocortisone and HWSP (Sample B) while Figure 1(b) is superimposed FTIR spectra of pure hydrocortisone (Sample A), CASP (Sample 2), and a (0.5 : 1) mixture of hydrocortisone and CASP (Sample C). Hydrocortisone has a broad O-H intermolecular hydrogen bonded peak at 3420 cm^−1^, weak olefinic or aromatic H-C= stretch at 3015 cm^−1^, merge C-H stretch at 2917 cm^−1^, five-member ring ketonic stretch at 1720 cm^−1^, six-member ring ketonic and enolic carbonyl stretch at 1665 cm^−1^, CH_2_ attached to ketonic of olefinic scissoring bend at 1433 cm^−1^, CH_2_CO scissoring vibration at 1408 cm^−1^ [31], and the fingerprint region which is distinct to every steroid. Pectin consists of a long chain polysaccharide with D-galacturonic acid as its monomer which resides in an alpha-(1-4) chain. As a polysaccharide, it has a broad carboxylic O-H stretch from 3500 to 2400 cm^−1^, a carbonyl C=O stretch at 1710 cm^−1^, an asymmetric and symmetric carboxylic anionic vibration at 1637 cm^−1^ and 1549 cm^−1^, a C-OH (carboxylic, ester, and alcoholic) stretch at 1047 cm^−1^ [32], and 1200–800 cm^−1^ for carbohydrates and in the fingerprint region.

From Figure 1, there is almost a complete overlap of hydrocortisone in HWSP mixture and hydrocortisone whilst some variation exists between hydrocortisone in CASP mixture and pure hydrocortisone. Hydrocortisone-CASP mixture has vibrations at 1576 cm^−1^ and fingerprint vibrations from 1160 to 438 cm^−1^ that coincide with pure hydrocortisone although with low intensities. This may be as a result of the small amount of hydrocortisone present in the mixture. In addition, hydrocortisone-CASP mixture has prominent and high intensity peaks at 3265 cm^−1^, 1564 cm^−1^, and 1045 cm^−1^ that are concurrent with peaks of CASP at these wavenumbers. This can be ascribed to the high concentration of pectin in the mixture. As the principal peaks in hydrocortisone also appear in all the mixtures of hydrocortisone and the CPH pectins, it can be inferred that there were no drug-excipient interactions.

**Figure 2:**
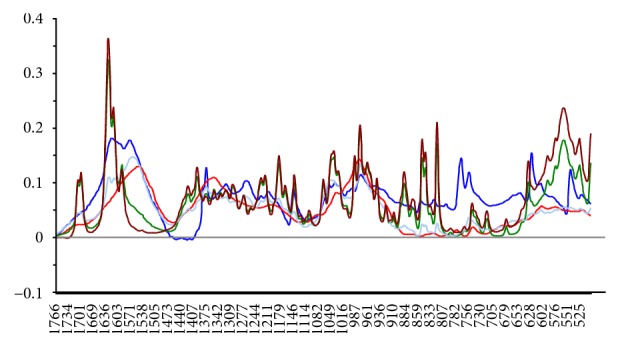
Savitzky-Golay second derivative spectra of selected wavelength of analysis (finger print region of 1776–500 cm^−1^).

**Figure 3:**
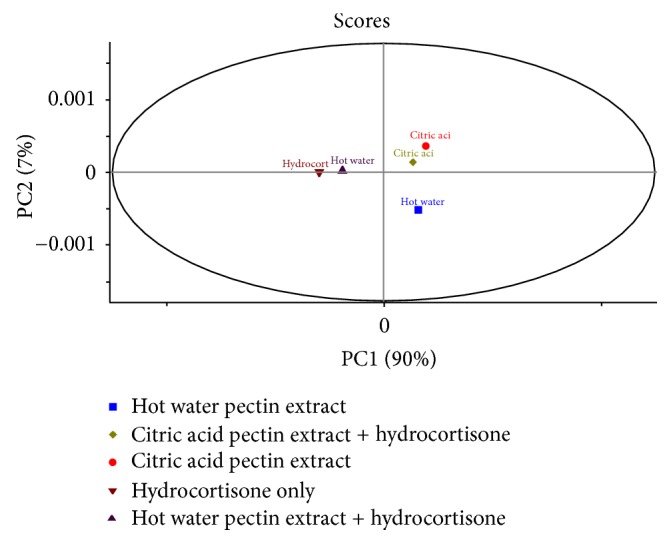
PCA plots of pure hydrocortisone (Sample A), HWSP (Sample 1), hydrocortisone in HWSP (Sample B), CASP (Sample 2), and hydrocortisone in CASP (Sample C).

**Figure 4:**
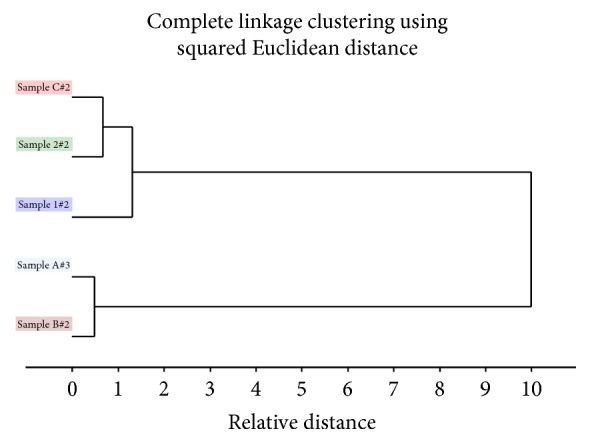
Hierarchical complete linkage cluster analysis with squared Euclidean distance of pure hydrocortisone (Sample A), HWSP (Sample 1), hydrocortisone in HWSP (Sample B), CASP (Sample 2), and hydrocortisone in CASP (Sample C).

Multivariate data analysis of FTIR spectra of the different pectin-based formulations and pure hydrocortisone was focused on pretreated second derivative spectra (Figure 2) of some prominent peaks and finger print regions (1776–500 cm^−1^) of hydrocortisone since the peak pattern at these wavenumbers is specific to every steroid and mostly employed in comparison studies [33]. PCA plots of pure hydrocortisone, hydrocortisone-HWSP, and hydrocortisone-CASP revealed that hydrocortisone-HWSP bears close relation with pure hydrocortisone, whereas hydrocortisone-CASP has some small variations from pure hydrocortisone (Figure 3). The spectral variations may be due to the CASP having absorption peaks with high intensities due to the high concentration of pectin in the mixture. From the PCA plot (Figure 3), PC1 was able to explain 90% of the variation in the spectra whereas PC2 explained 7% of the variation in the spectra. In all, 97% of the variations in all the spectra data were covered by 2PCs. Hierarchical complete linkage cluster analysis with squared Euclidean distance (Figure 4) gave credence to the fact that hydrocortisone-HWSP has close resemblance to pure hydrocortisone; thus HWSP forms a cluster with pure hydrocortisone whilst hydrocortisone-CASP has a distant relationship of about 10 relative distances from pure hydrocortisone.

### 3.2. Matrix Tablets Formulation and Drug Release Studies

Table 2 shows the physical properties of the hydrocortisone matrix tablet formulations. All the tablets exhibited good physical properties with average tablet weight (range: 300.0–300.8 mg; % deviation < 7.5%), friability (<1%), and hardness (>4 kg/cm^2^) within the acceptable compendial limits [34]. The content analysis of the hydrocortisone tablets yielded results in the acceptance criteria of 90–110% [29]. Hence the tablet formulations contained the requisite amounts of hydrocortisone and were of acceptable pharmaceutical quality.

**Figure 5:**
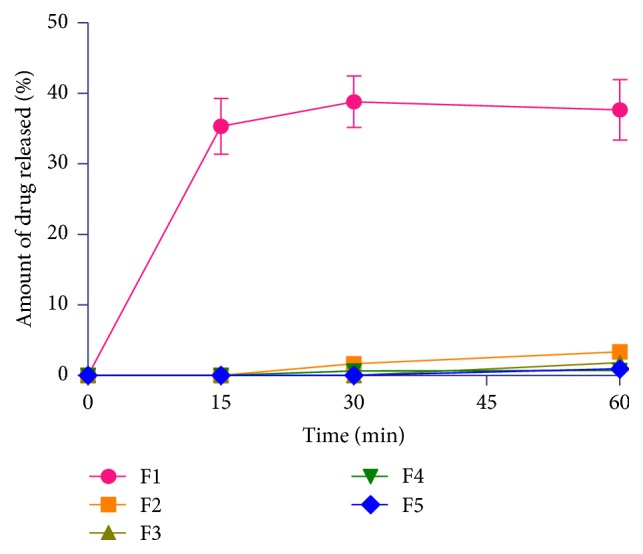
Dissolution profiles of uncoated pectin-free (F1) and CPH pectin-based (F2–F5) hydrocortisone tablet formulations in simulated gastric fluid (pH 1.2) (mean ± S. D*., n* = 3).

**Figure 6:**
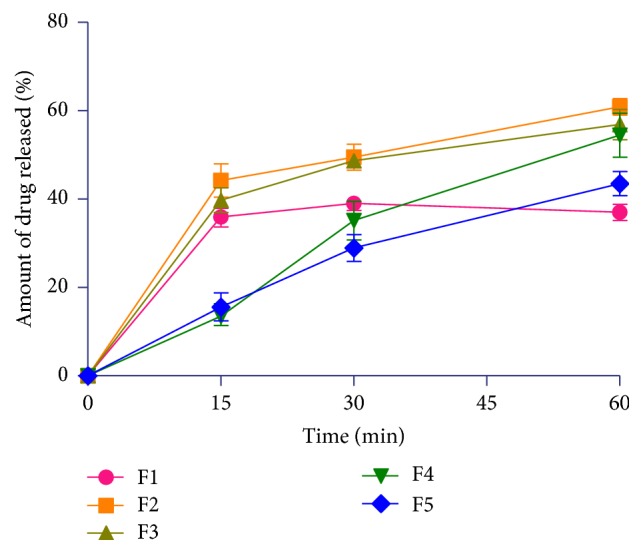
Dissolution profiles of uncoated pectin-free (F1) and CPH pectin-based (F2–F5) hydrocortisone tablet formulations in simulated small intestinal fluid (pH 6.8) (mean ± SEM*., n* = 3).

Hydrocortisone tablet formulations containing CPH pectin as release retardant were prepared for colonic release for use in adrenal insufficiency. The potential applications of colonic drug delivery systems include the chronotherapy of diseases with characteristic early morning symptoms due to their circadian rhythms [35–37].  Figure 5 presents the release profiles of the pectin-free and pectin-based hydrocortisone tablet formulations in simulated gastric fluid (pH 1.2). The amounts of hydrocortisone released in simulated gastric fluids (pH 1.2) at 60 min in formulations F1, F2, F3, F4, and F5 were 37.7%, 3.4%, 1.8%, 0.7%, and 0.9%, respectively. Formulations containing pectin exhibited extreme retarded drug release at pH 1.2 due to aggregation of the macromolecules in the acidic medium. On the other hand, the pectin-free formulation (F1) showed faster drug release in the acidic medium. In all the formulations which contained CPH pectin, the pectin served as a release retardant.  Figure 6 shows the release profiles of uncoated hydrocortisone matrix tablet formulations in simulated small intestinal fluid (pH 6.8). The* in vitro* release patterns of directly compressed immediate release hydrocortisone tablet (F1), a BCS class 2 drug, with slight water solubility, were lower than the release observed by Spireas and Bolton [38] who reported a 60% release in 30 min and 80% release in a liquisolid system with enhanced solubility in aqueous media. Even though formulation F1 contained a superdisintegrant as well as microcrystalline cellulose, with expected enhanced disintegrant properties, a release of 39.0% hydrocortisone in 30 min was observed. The dissolution rate of sparingly soluble drugs, such as hydrocortisone, prednisolone, and prednisone, is known to be poor and erratic, leading to therapeutic nonequivalence and variable bioavailability [38].

Controlled release profiles were demonstrated by the other uncoated hydrocortisone tablet formulations (F2–F5). The* in vitro* release profile of uncoated directly compressed hydrocortisone formulated with HWSP for modified release (F2) showed 60.9% release in 1 h. The directly compressed tablet formulated with CASP showed a slower release of 56.9% in 1 h (F3). The wet granulation formulations with HWSP (F4) and CASP (F5) showed a release of 54.5% and 43.5% in 1 h, respectively.

In both the simulated gastric (pH 1.2) and small intestinal (pH 6.8) fluids, the release profiles of the wet granulation tablet preparations (F4 and F5) showed greater retarded drug release compared to the directly compressed preparations (F2 and F3), even though the differences were not significant (*P* > 0.05). This shows that the method of granule formulation employed in the preparation of the tablets has some influence on drug release. Wet granulation enhances cohesion and formation of solid bridges and mechanical interlocking of the powder mix after solvent evaporation, thereby retarding drug release [39]. Wet granulation also improves powder properties through the coalescence of the primary particles with the liquid binder, thereby producing large particle sizes [40].

Drug release from CASP formulations also showed enhanced retarded drug release as compared to those containing HWSP (*P* > 0.05). Tablets containing citric acid release drug at a retarded rate due to suppression of polymer ionization as a result of a decrease in the microenvironmental pH [41, 42]. Pectin tends to aggregate as macromolecules in acidic media and deaggregate at neutral pH [43]. The release of drugs could be tailored with either of the CPH pectins to suit a particular modified release profile [44, 45]. The use of CPH pectin in the formulation of hydrocortisone matrix tablets exerted marked effect on drug release with reproducible release profiles.

**Figure 7:**
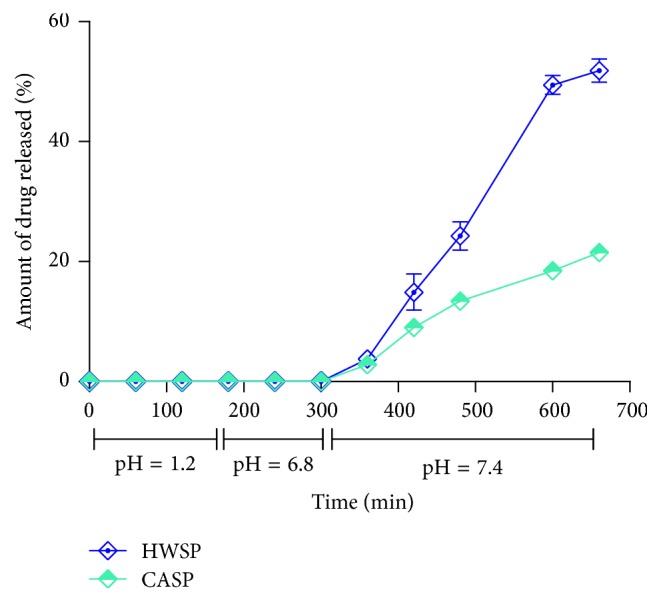
Dissolution profiles of film-coated wet granulation HWSP-based and CASP-based hydrocortisone matrix tablet formulations (~25% w/w) in simulated gastrointestinal fluids (pH 1.2, 2 h; pH 6.8, 3 h; pH 7.4, 6 h) (mean ± SEM*., n* = 3).

In this study, the drug delivery approach was to prevent hydrocortisone release in the stomach and small intestine and achieve a sudden drug release in the colon. The pH along the gastrointestinal tract is variable and pH-dependent polymers are employed to trigger the release of drugs in the colon. Time-dependent polymers also work on the principle of delaying drug release till the drug enters the colon. Colonic drug delivery could be optimized by combining pH-dependent and time-dependent polymers. The use of the two polymers would suppress drug release in the upper gastrointestinal tract while addressing the challenges of pH-dependent site specificity [46, 47]. The uncoated wet granulation tablet formulations of pectin (F4 and F5) exhibited greater control of drug release than the directly compressed formulations (F2 and F3) and were selected for coating as they could produce the desired suppression of drug release in the upper gastrointestinal tract. The pectin-based hydrocortisone matrix tablets were film-coated with Eudragit S 100 (outer coat) to confer acid resistance and HPMC (Methocel E50) (inner coat) to achieve timed release [48].  Figure 7 presents the release profiles of the film-coated tablets (25% w/w weight gain) in simulated gastrointestinal fluids. The two film-coated tablet formulations exhibited a lag phase of ~6 h, followed by a rapid hydrocortisone release for 6 h (*P* > 0.05), similar to the cortisol circadian rhythm. Even though a lag phase of ~6 h was observed, the coated formulation containing CASP exhibited too slow release, with 21.5% of hydrocortisone released in 5 h. However, tablets containing HWSP showed a release of 51.84% in 5 h after the ~6 h lag phase, hence selected as the optimized matrix tablet formulation.

The* in vitro* drug release data were fitted to zero order, first order, Higuchi, Hixson-Crowell, and Korsmeyer-Peppas kinetic models to determine the mechanisms of drug release [49]. The model with the highest correlation coefficient (*R*^2^) was selected as the model that best described the* in vitro* dissolution data (Table 3). The release kinetics of the directly compressed formulations F2 (*R*^2^ = 0.8803) and F3 (*R*^2^ = 0.9999) followed first order kinetics. This model describes drug release which is concentration dependent and is proportional to the amount of drug remaining in the dosage form. Drug release from formulation F4 followed the Korsmeyer-Peppas model (*R*^2^ = 0.9168, *n* = 0.4791). This model describes the release of drugs from polymeric systems. The mechanism of drug release was non-Fickian, as the *n* value was in the range 0.45 < *n* < 0.89 [50]. Drug release from F5 (*R*^2^ = 0.9859) and the film-coated formulations F6 (*R*^2^ = 0.9772) and F7 (*R*^2^ = 0.9826) followed the Higuchi kinetic model. Drug release from these systems is based on Fickian diffusion and is a function of the square root of time. This model provides sustained drug release within the therapeutic range and is suitable for the variable drug release patterns required in diseases such as adrenal insufficiency which are affected by circadian rhythm.

## 4. Conclusion

It can be concluded from the study that CPH pectin is compatible with hydrocortisone; hence the excipient can be used in formulations containing hydrocortisone without any possible drug-excipient interactions. Chemometric analysis of the FTIR spectra using Savitzky-Golay second derivative, PCA, and hierarchical complete linkage cluster analysis showed that hydrocortisone in HWSP bears close similarity with pure hydrocortisone while hydrocortisone in CASP showed a distant relationship of about 10 relative distances from pure hydrocortisone. The method of tablet production has influence on the release characteristics of hydrocortisone in aqueous medium as tablets produced by wet granulation had lower release rates than tablets produced by direct compression. The method of extraction of pectin extract from CPHs has effect on the release modifying properties of pectin. In this instance, CASP appeared to exhibit greater release modifying activity in aqueous medium than HWSP. It was shown that CPH pectin can be utilized to delay and control the release of hydrocortisone in oral formulations intended for delivery in the colon. Using CPH pectin, a lag phase of ~6 h was achieved for film-coated matrix tablets containing hydrocortisone, which was followed by rapid drug release in the colonic region in simulated gastrointestinal conditions, without enzymes. Oral matrix tablet formulations, incorporating CPH pectin and film-coated with HPMC for timed release and Eudragit for acid resistance, have potential use in controlling night time cortisol levels in patients suffering from adrenal insufficiency.

## Figures and Tables

**Table 1:** Formulation details of CPH pectin-based hydrocortisone matrix tablet formulations (~300 mg).

Code	Formulation technique	Hydrocortisone (mg)	CPH pectin (mg)	Microcrystalline cellulose (mg)	Dicalcium phosphate dihydrate (mg)	Magnesium stearate (mg)	Sodium starch glycolate (mg)
F1	Direct compression	10.0	-	111.2	166.8	3.0	9.0
F2	Direct compression	10.0	50.0^a^	118.5	118.5	3.0	-
F3	Direct compression	10.0	50.0^b^	118.5	118.5	3.0	-
F4	Wet granulation	10.0	50.0^a^	-	237.0	3.0	-
F5	Wet granulation	10.0	50.0^b^	-	237.0	3.0	-

a: hot water soluble pectin (HWSP); b: citric acid soluble pectin (CASP).

**Table 2:** Physical properties of CPH pectin-based hydrocortisone matrix tablets.

Code	Weight of tablets (mg)	Tablet thickness (mm)	Hardness (kg/cm^2^)	Friability (%)	Disintegration time (min)	Hydrocortisone content (%)^*∗*^
F1	300.0 ± 0.27	4.01 ± 0.003	11.4 ± 0.18	0.27	4.00	101.54 ± 0.34
F2	300.8 ± 0.50	4.00 ± 0.001	10.9 ± 0.18	0.33	ND	103.36 ± 0.44
F3	300.4 ± 0.32	4.00 ± 0.001	10.2 ± 0.03	0.41	ND	95.34 ± 0.33
F4	301.0 ± 0.32	3.55 ± 0.010	5.8 ± 0.05	0.40	ND	104.38 ± 0.34
F5	300.0 ± 0.54	3.47 ± 0.040	5.5 ± 0.05	0.60	ND	102.41 ± 0.13

ND: not determined for modified release formulations; ^*∗*^acceptance criteria: 90–110%.

**Table 3:** Kinetic parameters of CPH pectin-based hydrocortisone matrix tablets.

Code	Zero order		First order		Higuchi model		Hixson-Crowell		Korsmeyer-Peppas	
	*K*_0_	*R* ^2^	*K*_1_	*R* ^2^	*K*_H_	*R* ^2^	*K*_HC_	*R* ^2^	*n*	*R* ^2^
F2	13.9	0.8790	0.2796	0.8803	20.3	0.8134	0.3426	0.8798	0.1397	0.7288
F3	14.6	0.9987	0.2586	0.9999	22.1	0.9970	0.3304	0.9996	0.1849	0.9859
F4	20.1	0.8380	0.2722	0.8569	31.6	0.8993	0.3804	0.8506	0.4791	0.9168
F5	27.6	0.9564	0.3577	0.9713	42.6	0.9859	0.5073	0.9666	0.7868	0.9724
F6^*∗*^	3.5	0.9651	0.0405	0.9751	20.6	0.9772	0.0600	0.9720	3.0251	0.8570
F7^*∗∗*^	10.4	0.9826	0.1518	0.9807	59.7	0.9858	0.2059	0.9824	4.1138	0.9064

(a) ^*∗*^Film-coated HWSP-based hydrocortisone matrix tablets prepared by wet granulation (25% w/w weight gain); (b) ^*∗∗*^film-coated CASP-based hydrocortisone matrix tablets prepared by wet granulation (25% w/w weight gain); (c) *K*_0_, *K*_1_, *K*_HC_, *K*_H_ are kinetic constants for zero order, first order, Hixson-Crowell, and Higuchi models, respectively; *R*^2^ is the correlation coefficient; *n* is the release exponent.

## References

[1] Sajan J., Cinu T. A., Chacko A. J., Litty J., Jaseeda T. (2009). Chronotherapeutics and chronotherapeutic drug delivery systems. *Tropical Journal of Pharmaceutical Research*.

[2] Lin S.-Y., Kawashima Y. (2012). Current status and approaches to developing press-coated chronodelivery drug systems. *Journal of Controlled Release*.

[3] Ballesta A., Innominato P. F., Dallmann R., Rand D. A., Lévi F. A. (2017). Systems chronotherapeutics. *Pharmacological Reviews*.

[4] Wu J. C., Kelsoe J. R., Schachat C. (2009). Rapid and Sustained Antidepressant Response with Sleep Deprivation and Chronotherapy in Bipolar Disorder. *Biological Psychiatry*.

[5] Buttgereit F., Mehta D., Kirwan J. (2013). Low-dose prednisone chronotherapy for rheumatoid arthritis: A randomised clinical trial (CAPRA-2). *Annals of the Rheumatic Diseases*.

[6] Bornstein S. R., Allolio B., Arlt W. (2016). Diagnosis and treatment of primary adrenal insufficiency: an endocrine society clinical practice guideline. *The Journal of Clinical Endocrinology & Metabolism*.

[7] Elder C. J., Dimitri P. (2015). Hydrocortisone for adrenal insufficiency. *ADC - Education and Practice Edition*.

[8] Knutsson U., Dahlgren J., Marcus C. (1997). Circadian cortisol rhythms in healthy boys and girls: Relationship with age, growth, body composition, and pubertal development. *The Journal of Clinical Endocrinology & Metabolism*.

[9] Chan S., Debono M. (2010). Replication of cortisol circadian rhythm: new advances in hydrocortisone replacement therapy. *Therapeutic Advances in Endocrinology and Metabolism*.

[10] Debono M., Ross R. J. (2013). What is the best approach to tailoring hydrocortisone dose to meet patient needs in 2012?. *Clinical Endocrinology*.

[11] Lennernäs H., Skrtic S., Johannsson G. (2008). Replacement therapy of oral hydrocortisone in adrenal insufficiency: The influence of gastrointestinal factors. *Expert Opinion on Drug Metabolism & Toxicology*.

[12] Hindmarsh P. C., Charmandari E. (2015). Variation in absorption and half-life of hydrocortisone influence plasma cortisol concentrations. *Clinical Endocrinology*.

[13] Johannsson G., Nilsson A. G., Bergthorsdottir R. (2012). Improved cortisol exposure-time profile and outcome in patients with adrenal insufficiency: a prospective randomized trial of a novel hydrocortisone dual-release formulation. *The Journal of Clinical Endocrinology & Metabolism*.

[14] Falorni A., Minarelli V., Morelli S. (2013). Therapy of adrenal insufficiency: An update. *Endocrine Journal*.

[15] Johannsson G., Bergthorsdottir R., Nilsson A. G., Lennernas H., Hedner T., Skrtic S. (2009). Improving glucocorticoid replacement therapy using a novel modified-release hydrocortisone tablet: a pharmacokinetic study. *European Journal of Endocrinology*.

[16] Verma S., Vanryzin C., Sinaii N. (2010). A pharmacokinetic and pharmacodynamic study of delayed- and extended-release hydrocortisone (ChronocortTM) vs. conventional hydrocortisone (CortefTM) in the treatment of congenital adrenal hyperplasia. *Clinical Endocrinology*.

[17] Philip A. K., Philip B. (2010). Colon targeted drug delivery systems: a review on primary and novel approaches. *Oman Medical Journal*.

[18] Ofori-Kwakye K., Fell J. T., Sharma H. L., Smith A.-M. (2004). Gamma scintigraphic evaluation of film-coated tablets intended for colonic or biphasic release. *International Journal of Pharmaceutics*.

[19] Wu B., Chen Z., Wei X., Sun N., Lu Y., Wu W. (2007). Biphasic release of indomethacin from HPMC/pectin/calcium matrix tablet: I. Characterization and mechanistic study. *European Journal of Pharmaceutics and Biopharmaceutics*.

[20] Wong T. W., Colombo G., Sonvico F. (2011). Pectin matrix as oral drug delivery vehicle for colon cancer treatment. *AAPS PharmSciTech*.

[21] Sriamornsak P. (2011). Application of pectin in oral drug delivery. *Expert Opinion on Drug Delivery*.

[22] Newton A. M. J., Kaur B., Indiana V. L., Rajesh K. S. (2014). Chronotherapeutic drug delivery of pectin vs guar gum, xanthan gum controlled release colon targeted directly compressed propranolol HCl matrix tablets. *SAJ Pharmacy and Pharmacology*.

[23] Vriesmann L. C., Teófilo R. F., Lúcia de Oliveira Petkowicz C. (2012). Extraction and characterization of pectin from cacao pod husks (*Theobroma cacao* L.) with citric acid. *LWT - Food Science and Technology*.

[24] Chan S.-Y., Choo W.-S. (2013). Effect of extraction conditions on the yield and chemical properties of pectin from cocoa husks. *Food Chemistry*.

[25] Adi-Dako O., Ofori-Kwakye K., Frimpong Manso S., Boakye-Gyasi M. E., Sasu C., Pobee M. (2016). Physicochemical and antimicrobial properties of cocoa pod husk pectin intended as a versatile pharmaceutical excipient and nutraceutical. *Journal of Pharmaceutics*.

[26] Adi-Dako O., Oppong Bekoe S., Ofori-Kwakye K., Appiah E., Peprah P. (2017). Novel HPLC analysis of hydrocortisone in conventional and controlled-release pharmaceutical preparations. *Journal of Pharmaceutics*.

[27] Gazzaniga A., Palugan L., Foppoli A., Sangalli M. E. (2008). Oral pulsatile delivery systems based on swellable hydrophilic polymers. *European Journal of Pharmaceutics and Biopharmaceutics*.

[28] Akhgari A., Sadeghi F., Garekani H. A. (2006). Combination of time-dependent and pH-dependent polymethacrylates as a single coating formulation for colonic delivery of indomethacin pellets. *International Journal of Pharmaceutics*.

[29] United States Pharmacopoeia and National Formulary (2013). *United States Pharmacopoeia XXIII*.

[30] Verma R. K., Garg S. (2005). Selection of excipients for extended release formulations of glipizide through drug-excipient compatibility testing. *Journal of Pharmaceutical and Biomedical Analysis*.

[31] Boul A. D., Blunt J. W., Browne J. W. (1971). Microbiological hydroxylation of steroids. Part II. Structural information and infrared spectrometry: Carbonyl, perturbed methylene, and hydroxy-vibrations of steroidal ketones and alcohols. *Journal of the Chemical Society C: Organic*.

[32] Seslija S., Veljovic D., Kalagasidis Krusic M., Stevanovic J., Velickovic S., Popovic I. (2016). Cross-linking of highly methoxylated pectin with copper: The specific anion influence. *New Journal of Chemistry*.

[33] Dobringer K., Katzennellenbogan E. R., Jones R. N. (1953). *Infrared Absorption Spectra of Steroids*.

[34] British Pharmacopoeia (2013). *British Pharmacopoeia Commission*.

[35] Wei X., Sun N., Wu B., Yin C., Wu W. (2006). Sigmoidal release of indomethacin from pectin matrix tablets: effect of in situ crosslinking by calcium cations. *International Journal of Pharmaceutics*.

[36] Singh B. N. (2007). Modified-release solid formulations for colonic delivery. *Recent Patents on Drug Delivery & Formulation*.

[37] Sreelatha D., Brahma C. K. (2013). Colon targeted drug delivery—a review on primary and novel approaches. *Journal of Global Trends in Pharmaceutical Sciences*.

[38] Spireas S., Bolton S. M. Liquisolid systems and methods of preparing same.

[39] McConville J. T., Ross A. C., Chambers A. R., Smith G., Florence A. J., Stevens H. N. E. (2004). The effect of wet granulation on the erosion behaviour of an HPMC-lactose tablet, used as a rate-controlling component in a pulsatile drug delivery capsule formulation. *European Journal of Pharmaceutics and Biopharmaceutics*.

[40] Nguyen T. H., Shen W., Hapgood K. (2010). Effect of formulation hydrophobicity on drug distribution in wet granulation. *Chemical Engineering Journal*.

[41] Nykänen P., Lempää S., Aaltonen M.-L., Jürjenson H., Veski P., Marvola M. (2001). Citric acid as excipient in multiple-unit enteric-coated tablets for targeting drugs on the colon. *International Journal of Pharmaceutics*.

[42] Bruce L. D., Shah N. H., Waseem Malick A., Infeld M. H., McGinity J. W. (2005). Properties of hot-melt extruded tablet formulations for the colonic delivery of 5-aminosalicylic acid. *European Journal of Pharmaceutics and Biopharmaceutics*.

[43] Fan L.-F., He W., Bai M. (2008). Biphasic drug release: Permeability and swelling of pectin/ethylcellulose films, and in vitro and in vivo correlation of film-coated pellets in dogs. *Chemical & Pharmaceutical Bulletin*.

[44] Singh A. V. (2011). Biopolymers in drug delivery: a review. *Pharmacology Online Newsletters*.

[45] Chaudhari P. S., Shanmugasundaram P. (2014). Modification & characterization of natural polymer for development of dosage forms. *International Journal of PharmTech Research*.

[46] Qi M., Wang P., Wu D. (2003). A novel pH- and time-dependent system for colonic drug delivery. *Drug Development and Industrial Pharmacy*.

[47] Sangalli M. E., Maroni A., Zema L., Busetti C., Giordano F., Gazzaniga A. (2001). *In vitro* and *in vivo* evaluation of an oral system for time and/or site-specific drug delivery. *Journal of Controlled Release*.

[48] Cao Q.-R., Choi H.-G., Kim D.-C., Lee B.-J. (2004). Release behavior and photo-image of nifedipine tablet coated with high viscosity grade hydroxypropylmethylcellulose: Effect of coating conditions. *International Journal of Pharmaceutics*.

[49] Costa P., Sousa Lobo J. M. (2001). Modeling and comparison of dissolution profiles. *European Journal of Pharmaceutical Sciences*.

[50] Korsmeyer R. W., Gurny R., Doelker E., Buri P., Peppas N. A. (1983). Mechanisms of solute release from porous hydrophilic polymers. *International Journal of Pharmaceutics*.

